# Supplementary material for: Metabolic influence of core ciliates within the rumen microbiome
Source: ISME J. 2023 May 11;17(7):1128–40. doi: 10.1038/s41396-023-01407-y (PMC10284877; doi:10.1038/s41396-023-01407-y)
Supplement: Supplementary file 1 — Supplementary Figure S1 [file 41396_2023_1407_MOESM1_ESM.pdf]

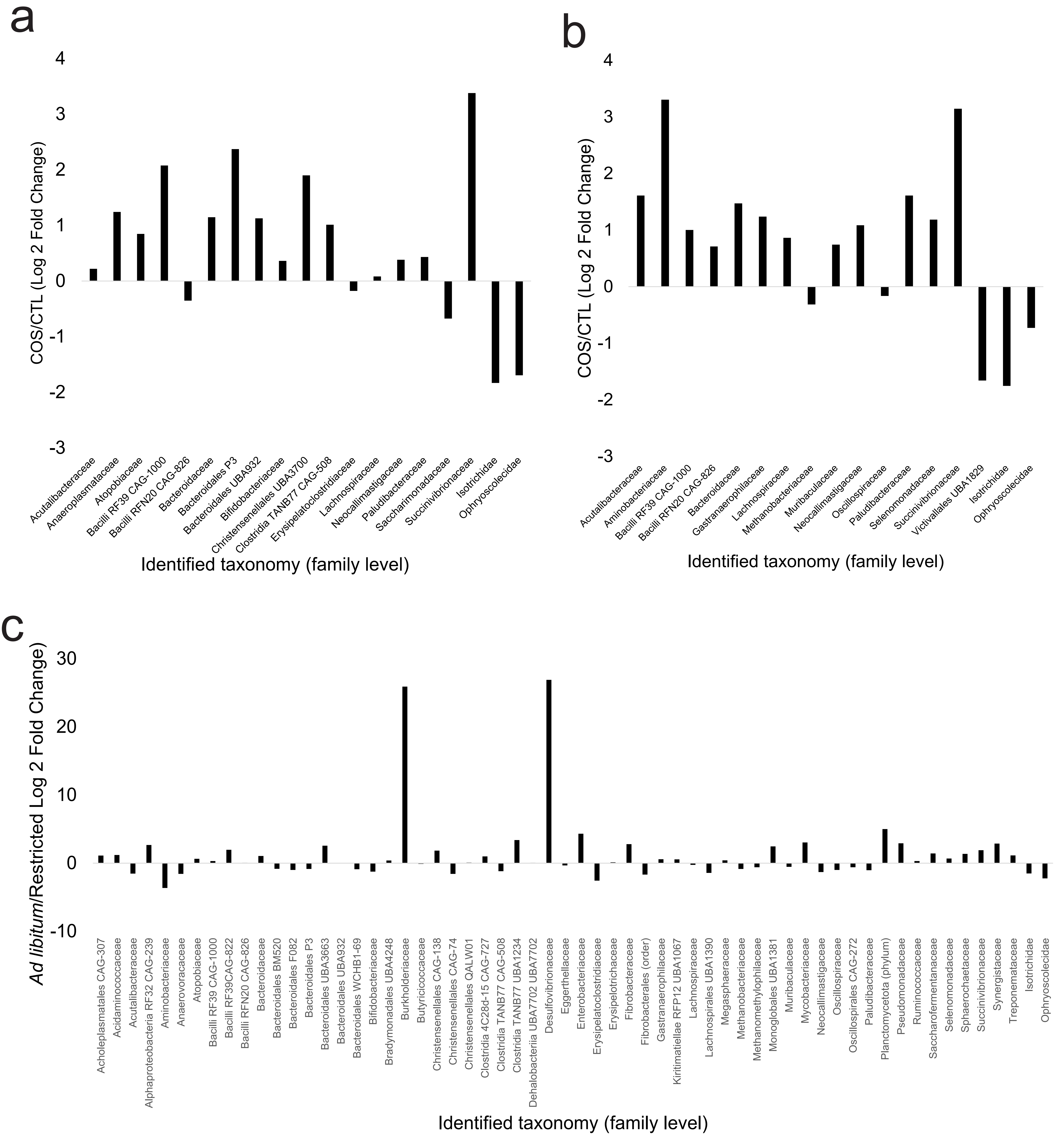

Supplementary Figure S1. Protein abundance level ratios calculated for commonly observed families between animals fed high/ low starch diets. Log 2-Fold change ratios between average LFQ intensities for dairy cattle (a), goats (b) or beef cattle from an independent dataset (c) fed high or low starch diets. Positive ratios show higher average protein abundance level for a given taxonomic family in animals that were fed high starch diets (COS or ad libitum). Negative ratios show higher average protein abundance levels for a given taxonomic family in animals that were fed the low starch diets (CTL or Restricted). Rarely observed families with < 2 MAGs with detected proteins across both conditions were excluded from the analysis. All ratios, and subsequent correlation tests are available in **Supplementary Table S9**.
